# Echocardiographic integrated backscatter for detecting progression and regression of aortic valve calcifications in rats

**DOI:** 10.1186/1476-7120-11-4

**Published:** 2013-01-26

**Authors:** Bram Roosens, Gezim Bala, Kris Gillis, Isabel Remory, Steven Droogmans, Joan Somja, Eléonore Delvenne, Joeri De Nayer, Johan Schiettecatte, Philippe Delvenne, Patrizio Lancellotti, Guy Van Camp, Bernard Cosyns

**Affiliations:** 1 Department of Cardiology, Centrum Voor Hart- en Vaatziekten (CHVZ), UZ Brussel, Laarbeeklaan 101, B-1090, Brussels, Belgium; 2 In Vivo Cellular and Molecular Imaging (ICMI), Faculty of Medicine and Pharmacy, Vrije Universiteit Brussel (VUB), Laarbeeklaan 103, B-1090, Brussels, Belgium; 3 Department of Pathology, University of Liège, CHU Sart Tilman, B-4000, Liège, Belgium; 4 Laboratory of Clinical Chemistry & Radioimmunology, UZ Brussel, Laarbeeklaan 101, B-1090, Brussels, Belgium; 5 GIGA Cardiovascular Sciences, Heart Valve Clinic, University of Liège, CHU Sart Tilman, B-4000, Liège, Belgium

**Keywords:** Aortic valve calcifications, Calcific aortic valve disease, Echocardiography, Integrated backscatter, Renal failure, Small animal

## Abstract

**Background:**

Calcification is an independent predictor of mortality in calcific aortic valve disease (CAVD). The aim of this study was to evaluate the use of non-invasive, non-ionizing echocardiographic calibrated integrated backscatter (cIB) for monitoring progression and subsequent regression of aortic valvular calcifications in a rat model of reversible renal failure with CAVD, compared to histology.

**Methods:**

28 male Wistar rats were prospectively followed during 21 weeks. Group 1 (N=14) was fed with a 0.5% adenine diet for 9 weeks to induce renal failure and CAVD. Group 2 (N=14) received a standard diet. At week 9, six animals of each group were killed. The remaining animals of group 1 (N=8) and group 2 (N=8) were kept on a standard diet for an additional 12 weeks. cIB of the aortic valve was calculated at baseline, 9 and 21 weeks, followed by measurement of the calcified area (Ca Area) on histology.

**Results:**

At week 9, cIB values and Ca Area of the aortic valve were significantly increased in the adenine-fed rats compared to baseline and controls. After 12 weeks of adenine diet cessation, cIB values and Ca Area of group 1 decreased compared to week 9, while there was no longer a significant difference compared to age-matched controls of group 2.

**Conclusions:**

cIB is a non-invasive tool allowing quantitative monitoring of CAVD progression and regression in a rat model of reversible renal failure, as validated by comparison with histology. This technique might become useful for assessing CAVD during targeted therapy.

## Background

Calcific aortic valve disease (CAVD) is currently the most frequent valvular heart disease in the western world [1]. The extent of calcification has been shown to be an independent predictor of morbidity and mortality in CAVD [2,3]. Medical therapies to attenuate or even prevent progression of CAVD are being increasingly investigated [4]. Evaluation of medication-induced reversibility of valvular calcifications with non-invasive imaging modalities is promising. Computed tomography (CT) is considered as the reference technique for the quantification and follow-up of calcifications in patients at increased cardiovascular risk [5]. However, cardiac CT raises concerns about irradiation, which may limit repetitive scanning [6]. Recently, we have validated the non-invasive and non-ionizing technique of echocardiographic integrated backscatter (IB) for the quantification of both normal, age-related and vitamin D-induced aortic valve calcifications in rats [7,8]. Animal models such as rats are increasingly used to study the pathophysiology of CAVD and to test potential medical therapies [9]. In that perspective, echocardiographic IB might be of interest to monitor the progression and regression of aortic valve calcifications in animals and humans.

Rats fed with an adenine and high phosphate diet are a validated model of renal failure and secondary hyperparathyroidism with subsequent CAVD. A previous study has shown that discontinuation of such a diet led to progressive recovery of the renal function and spontaneous regression of aortic valve calcifications [10].

The aim of the present study was to evaluate the use of echocardiographic IB as a non-invasive and non-ionizing imaging technique for monitoring progression and subsequent regression of aortic valvular calcifications in a validated rat model of reversible renal failure with CAVD, compared to histology.

## Methods

### Study design

Twenty-eight male Wistar Unilever rats (Harlan, The Netherlands, 7 weeks old) were prospectively divided in two groups and followed for 21 weeks. Group 1 (N=14) was fed with a 0.5% adenine, 1.5% phosphorus, 0.6% calcium diet (ssniff Spezialdiaten GmbH, Soest, Germany) for 9 weeks to induce renal failure. Group 2 (N=14) of control rats was fed with a standard diet (rat maintenance diet, SAFE, France). At week 9, six animals of each group were killed for histopathological analysis. For the regression phase, all remaining animals of group 1 (N=8) and group 2 (N=8) were kept on a standard diet and followed for an additional 12 weeks. At baseline, week 9 and week 21, echocardiography was performed and a blood sample was taken. At the end of the study the remaining animals were killed with 120 mg/Kg sodium pentobarbital intravenously. The hearts were excised within 30 minutes of death and fixed in neutral buffered formalin 4% for 24 hours, followed by histology. The observers were blinded to the group identity for all analysis. The Ethics Committee for animal studies of the Vrije Universiteit Brussel approved the study protocol. Guidelines of the National Institute of Health’s Guide for the Care and Use of Laboratory Animals (NIH publication 86–23, revised 1985) were followed.

### Animal handling

During the whole study, all rats were housed in stainless steel cages with sawdust bedding. They were kept at a twelve hour day/night cycle, an average room temperature of 24°C and a relative humidity of 50%. Food and water were provided ad libitum.

### Physiological parameters

Body weight was determined weekly. Physical condition of the animals was assessed daily.

### Echocardiography

Rats were anaesthetized with 5% isoflurane gas (S.A. Abbott N.V., Ottignies, Belgium). Subsequently, the animals were put under continuous isoflurane gas (2.5%) anaesthesia with 2L O_2_/min as carrier gas. The anterior chest wall was shaved and the rat was placed in left lateral decubitus on a wooden bench in order to obtain optimal image quality and views, as previously described [11]. Electrocardiogram electrodes were fixed on the paws. A 10 MHz neonatal probe (10S) operated a 10 MHz was used, connected to a Vivid 7 Pro system (GE Medical Systems, Milwaukee, WI, USA).

#### Left ventricular and aortic valve function

As previously described [11], left ventricular (LV) volumes were calculated from the M-mode tracings in the short-axis view (papillary muscle level, average of 3 cycles) using an ellipsoid model: Pi × D^3^/3 (where D is the diameter of the ventricle in the short-axis view). LV ejection fraction (EF) was calculated from the LV end-diastolic volume (LVEDV) and LV end-systolic volume (LVESV): LVEF=([(LVEDV – LVESV)/LVEDV] × 100). Heart rate was determined from the R-R interval on the electrocardiogram. Cardiac output (CO) was calculated as stroke volume × heart rate, with stroke volume determined from the LV EDV - LV ESV. The peak diastolic early (E) and late (A) velocities were assessed from the mitral valve pulsed wave Doppler inflow pattern in the apical 4-chamber view. The deceleration time (Dec Time) was measured. The parasternal long axis view was used to measure the diameter of the LV outflow tract (LVOT). The aortic valve (AV) area was calculated using the continuity equation: [LVOT (TVI) × LVOT area] / Aortic (TVI). Mean and peak transaortic valve pressure gradients (Aortic Mean PG, Aortic Peak PG) were measured in the apical 5-chamber view.

#### Integrated backscatter analysis

IB of tissue ultrasound reflectivity, measured in decibels (dB), was used for the quantification of aortic valve calcifications [8]. IB is the integrated average power of the squared radiofrequency component of the reflected backscatter signal from a region of interest (ROI) within the tissue [12]. Two-dimensional standard transthoracic parasternal short- and long-axis ultrasound images were obtained at a constant angle of insonification in all rats. Control settings such as image zoom, focus position, frame rate, lateral- and time-gain compensation were kept constant for every measurement to obtain uniform brightness and to facilitate reproducible sampling of backscatter values [13]. IB values were acquired and averaged over 3 cardiac cycles off-line with a tissue quantification analysis package (Echopac 110.0.0 (BT10), GE Vingmed) by placing fifteen 1×1-mm circle-shaped sample volumes (ROIs) on the aortic valve in all images. Moreover, global ROIs I and II were placed around the whole aortic valve in respectively the short- and long-axis. The positions of the ROIs were within the same region for all animals and during all cardiac cycles. Blood pools (BP): BP1 of the atrial cavity in the short-axis, BP2 of the LV outflow tract in the long-axis. Calibration of the integrated backscatter values was done to compensate for the attenuation of the backscatter signal due to intervening structures between the skin surface and the ROIs [14]. Calibrated IB (cIB) was determined by subtracting IB values from the tissue with the blood pool IB values adjacent to the tissue (cIB value (ROI X) = [IB value (ROI X) – IB value (ROI BPX)]). An increase in cIB values reflects an increase in echogenicity. Mean cIB values for each structure were calculated as follows: cIB value (ROI_AVSA_ (aortic valve, short-axis)) = Mean cIB value (ROI 1 to 10); cIB value (ROI_AVLA_ (aortic valve, long-axis)) = Mean cIB value (ROI 11 to 15); cIB value (ROI_AVTOT_ (aortic valve, total)) = Mean cIB value (ROI_AVSA_, ROI_AVLA_); cIB value (ROI_AVGLOB_ (aortic valve, global)) = Mean cIB value (ROI I, II) [8].

### Histopathology

The hearts were cut in an axial plane (from base to apex) and embedded in paraffin. Nine ranges of sections were made from each paraffin embedded heart, with approximately 50 μm between each range in order to visualize a maximum amount of tissue. Slices of 4–6 μm thick were stained with classic haematoxylin-eosin and Von Kossa. Experienced pathologists performed tissue analysis with a PC digital image camera (Digital Sight DS-5M, Nikon Corp, Japan) mounted on an Axiolab Zeiss light microscope (Carl Zeiss Corp, Germany). The NIH Image program (Image-J 1.47a, National Institutes of Health, Bethesda, MD, USA) was used to calculate the aortic valve calcified area (Ca Area, mm^2^) for each rat, by summation of areas of deposits in the most calcified slice. The program was calibrated with a graduated slice.

### Biochemistry

Blood samples were acquired from a tail vein in all rats before echocardiography. Plasma was analyzed for creatinine, blood urea nitrogen (BUN), total calcium (Ca), phosphorus (P), alkaline phosphate (AP) and osteocalcin by using reagent kits according to the manufacturer’s instructions. The calcium × phosphorus (Ca × P) product was calculated.

### Statistical analysis

Data are expressed as mean ± standard error of the mean. Variables were tested for normality of distribution and homogeneity of variance by means of the Shapiro-Wilk and Levene tests. Comparisons between groups were performed by using the unpaired and paired Student t-test. The Mann–Whitney U and Wilcoxon test were used for variables approximating normality of distribution. Study variability and correlations between continuous variables were evaluated by means of the Pearson correlation coefficient. All p values were calculated two-tailed. A p value < 0.05 was considered significant. Statistical analysis was done with IBM SPSS Statistics (version 20.0.0, SPSS Inc., an IBM Company, Chicago, USA).

## Results

### Body weight

At baseline there was no significant difference in mean body weight between groups (group 1: 293 ± 5 g; group 2: 295 ± 5 g; p = 0.775). At week 9, mean body weight significantly decreased in the adenine-fed group 1 (252 ± 11 g) compared to control group 2 (468 ± 7 g; p < 0.001). After adenine-diet cessation, animals from group 1 regained weight (week 21, group 1: 481 ± 18 g; group 2: 560 ± 13 g; p = 0.003).

### Echocardiography

#### Left ventricular and aortic valve function

There were no significant differences between groups in LV and aortic valve parameters at baseline. In the adenine-fed group 1, there was a significant decrease in EF, CO and transvalvular PG at week 9 compared to baseline and group 2. After adenine-diet cessation, the EF, CO and PG recovered by week 21 in group 1. During the study course there was no significant difference in LV diastolic function (E/A, Dec Time) and aortic valve area between groups (Table 1).

**Table 1:** Echocardiographic evaluation for each group

	**Baseline**			**Week 9**			**Week 21**		
	Gr 1 (N=14)	Gr 2 (N=14)	p (Gr 1 vs. Gr 2)	Gr 1 (N=14)	Gr 2 (N=14)	p (Gr 1 vs. Gr 2)	Gr 1 (N=8)	Gr 2 (N=8)	p (Gr 1 vs. Gr 2)
	Adenine 0.5%	Controls		Adenine 0.5%	Controls		Adenine 0.5%	Controls	
EF (%)	83 ± 2	84 ± 1	0.690	67 ± 3 **	75 ± 1 **	0.013	78 ± 1 *#	74 ± 3 *	0.328
CO (ml/min)	171 ± 11	163 ± 11	0.616	95 ± 6 **	185 ± 6 *	< 0.001	214 ± 18 #	207 ± 7	0.738
E/A	1.72 ± 0.18	1.61 ± 0.07	0.970	1.53 ± 0.06	1.52 ± 0.05	0.881	1.48 ± 0.09	1.51 ± 0.07	0.787
Dec time (ms)	44 ± 2	43 ± 1	0.707	45 ± 3	42 ± 2	0.522	47 ± 2	42 ± 2	0.103
AV Area (mm^2^)	5.1 ± 0.2	4.6 ± 0.3	0.217	5.1 ± 0.4	6.0 ± 0.4 *	0.095	9.5 ± 1.2 *#	8.2 ± 0.7 *#	0.505
Aortic Peak PG (mmHg)	1.77 ± 0.13	1.64 ± 0.10	0.329	0.97 ± 0.08 *	1.48 ± 0.17	0.020	1.46 ± 0.16 #	1.67 ± 0.12	0.317
Aortic Mean PG (mmHg)	1.30 ± 0.10	1.17 ± 0.07	0.376	0.68 ± 0.05 *	1.02 ± 0.11	0.008	1.17 ± 0.08 #	1.18 ± 0.09	0.925

Data are represented as means ± SEM. AV, aortic valve; CO, cardiac output; Dec Time, deceleration time; EF, ejection fraction; Gr., group; PG, pressure gradient. * Vs. Baseline; p < 0.05. ** Vs. Baseline; p < 0.001. # Vs. Week 9; p < 0.05.

#### Integrated backscatter analysis of echocardiography

**Figure 1:**
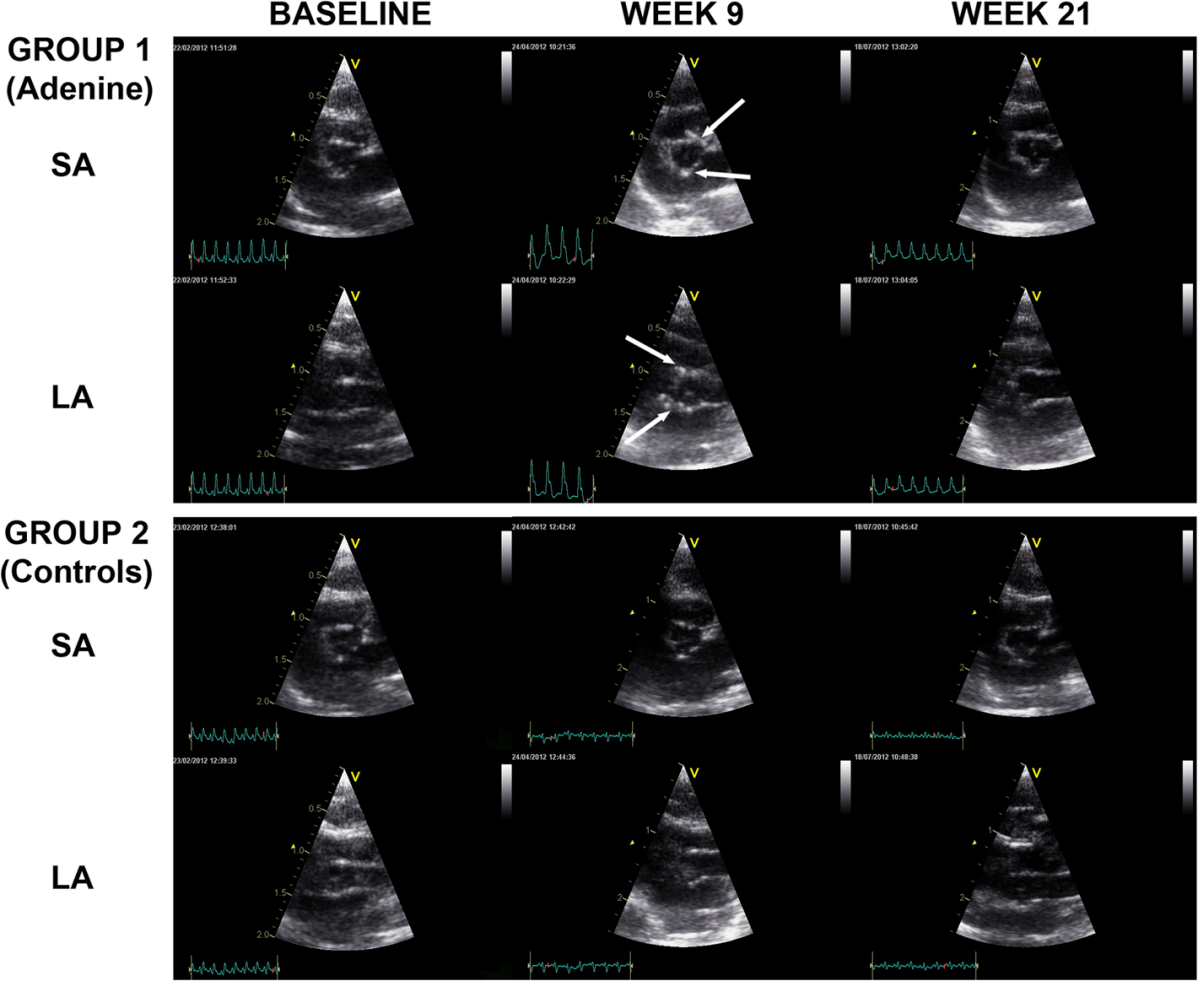
**Echocardiography of the aortic valve of adenine-fed and control rats.** Echocardiography of the aortic valve in the short-axis (SA) and long-axis (LA) of adenine-fed group 1 and control group 2 rats, at baseline, week 9 and week 21. Arrows indicate valvular calcifications. After cessation of the adenine-diet in group 1 (after week 9), regression of calcifications is observed.

There was a significant correlation between the global cIB values (ROI_AVGLOB_) and the mean cIB values (ROI_AVTOT_) of the aortic valve (Pearson r = 0.83; p < 0.001). At baseline, there were no significant differences in cIB values between groups. After 9 weeks, the cIB values of the aortic valve were significantly increased in the adenine-fed group 1 compared to baseline and control group 2. After adenine diet-cessation at the end of the study, the cIB values of group 1 decreased compared to week 9, and there was no more significant difference compared to controls of the same age (Table 2, Figure 1). There was a significant intra- and inter-observer reproducibility of cIB values for ROI_AVGLOB_ (respectively Pearson r = 0.736; p = 0.037; and Pearson r = 0.676; p = 0.032) and ROI_AVTOT_ (respectively Pearson r = 0.725; p = 0.042; and Pearson r = 0.652; p = 0.041).

**Table 2:** Calibrated integrated backscatter (cIB) values and histological calcium area (Ca Area) of the aortic valve

		**Gr 1**	**Gr 2**	**p (Gr 1 vs. Gr 2)**
		Adenine 0.5%	Controls	
cIB Baseline (dB)	ROI_AVTOT_	13.4 ± 0.4	14.2 ± 0.5	0.259
	ROI_GLOB_	16.9 ± 0.6	16.6 ± 0.6	0.739
cIB Week 9(dB)	ROI_AVTOT_	16.4 ± 0.5 **	13.3 ± 0.6	0.001
	ROI_GLOB_	20.7 ± 0.5 **	17.2 ± 0.7	< 0.001
cIB Week 21 (dB)	ROI_AVTOT_	14.8 ± 0.8	14.2 ± 0.5	0.531
	ROI_GLOB_	18.3 ± 1.3	18.7 ± 1.0	0.784
Ca Area Week 9 (mm^2^)		0.076 ± 0.021	0.0 ± 0.0	0.005
Ca Area Week 21 (mm^2^)		0.023 ± 0.012 #	0.0 ± 0.0	0.074

Data are represented as means ± SEM. dB, decibel; Gr., group; ROI_AVTOT_, region of interest, aortic valve, total; ROI_GLOB_, region of interest, aortic valve, global. ** Vs. Baseline; p < 0.001. # Vs. Week 9; p = 0.037.

### Histology

**Figure 2:**
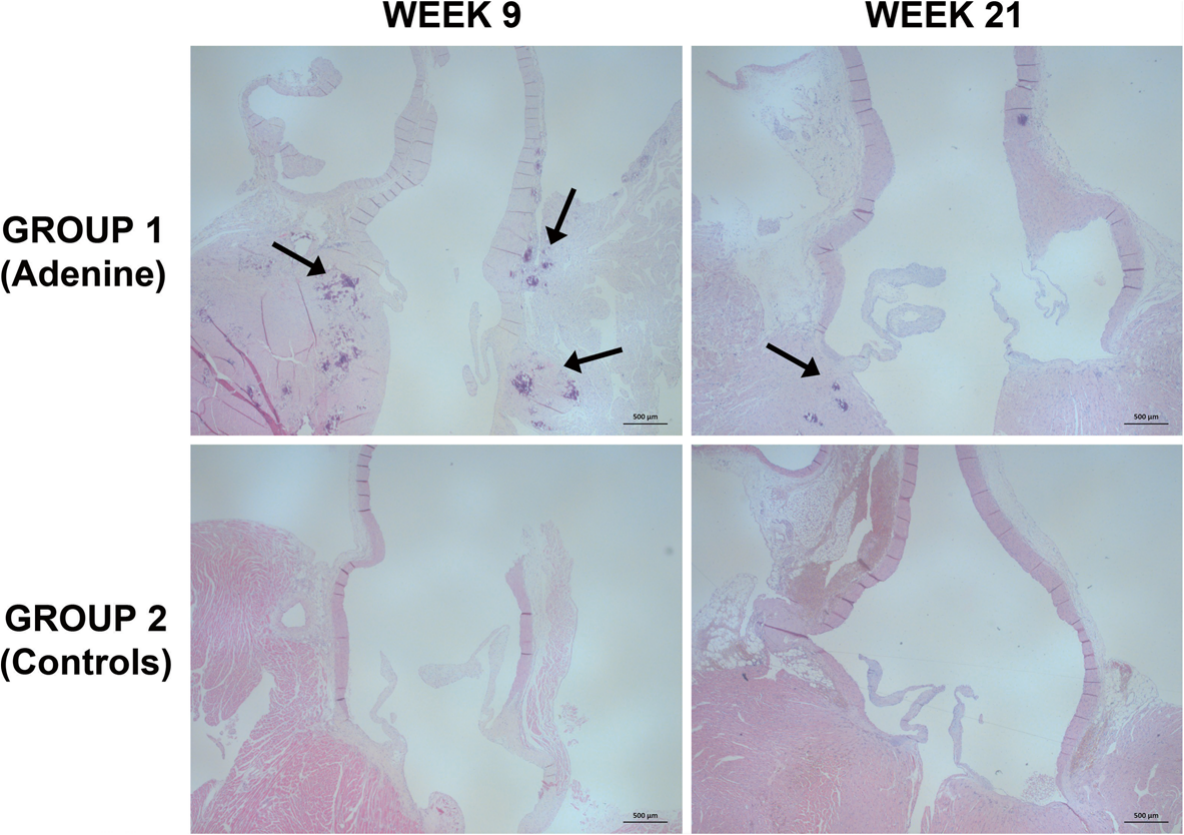
**Histology of the aortic valve of adenine-fed and control rats.** Histology of the aortic valve of adenine-fed group 1 and control group 2 rats, at weeks 9 and week 21. Arrows indicate valvular calcifications that are mainly situated in the aortic annulus. After cessation of the adenine-diet in group 1 (after week 9), regression of calcifications is observed. Scale bar is 500 μm, original magnification at 25 x, haematoxylin-eosin staining.

At week 9, the Ca Area of the aortic valve was significantly higher in the adenine-fed group 1 compared to control group 2. At the end of the study, the Ca Area of group 1 significantly decreased compared to week 9 and there was no more significant difference compared to age-matched controls (Table 2, Figure 2). There were significant correlations between cIB values and the histological Ca Area for all rats (respectively ROI_AVGLOB_: Pearson r = 0.546; p = 0.003; and ROI_AVTOT_: Pearson r = 0.487; p = 0.010 ).

### Blood Biochemistry

At baseline, there was no significant difference in blood biochemistry between groups. After 9 weeks, there was a significant increase in creatinine, BUN, P, and Ca × P in the adenine-fed group 1 compared to control group 2 and baseline. At week 9, AP and osteocalcin were also significantly increased in the adenine-fed group 1 compared to control group 2.

After 21 weeks, the renal function slightly recovered in group 1 after adenine diet discontinuation. There were no more significant differences between groups for P, Ca × P, and AP. However, osteocalcin remained significantly increased in group 1 compared to group 2 (Table 3).

**Table 3:** Blood biochemistry values for each group

	**Baseline**			**Week 9**			**Week 21**		
	Gr 1 (N=14)	Gr 2 (N=14)	p (Gr 1 vs. Gr 2)	Gr 1 (N=14)	Gr 2 (N=14)	p (Gr 1 vs. Gr 2)	Gr 1 (N=8)	Gr 2 (N=8)	p (Gr 1 vs. Gr 2)
	Adenine 0.5%	Controls		Adenine 0.5%	Controls		Adenine 0.5%	Controls	
Creatinine (mg/dl)	0.39 ± 0.01	0.42 ± 0.01	0.078	2.33 ± 0.16 *	0.47 ± 0.01 *	< 0.001	2.01 ± 0.36 *	0.47 ± 0.01 *	< 0.001
BUN (mg/dl)	34.7 ± 1.7	33.2 ± 1.2	0.466	240.6 ± 13.0 *	33.4 ± 1.0	< 0.001	182.2 ± 28.0 *	32.3 ± 1.0	< 0.001
Ca (mg/dl)	10.6 ± 0.1	10.3 ± 0.2	0.478	7.8 ± 0.2 *	10.1 ± 0.1	< 0.001	10.0 ± 0.3 *#	10.7 ± 0.1 #	0.028
P (mg/dl)	6.6 ± 0.3	7.0 ± 0.2	0.322	17.0 ± 0.9 *	5.6 ± 0.3 *	< 0.001	6.7 ± 1.2 #	5.0 ± 0.2 *	0.234
Ca x P (mg2/dl2)	70.0 ± 3.2	72.1 ± 2.9	0.639	132.8 ± 9.2 *	56.4 ± 1.8 *	< 0.001	65.4 ± 9.4 #	52.1 ± 1.5 *	0.234
AP (U/L)	315.5 ± 9.8	303.6 ± 13.0	0.473	171.5 ± 6.5 **	133.1 ± 7.0 **	0.001	117.5 ± 2.9 **##	115.6 ± 5.6 **##	0.770
Osteocalcin	58.5 ± 2.2	54.2 ± 1.8	0.082	62.1 ± 2.2	19.8 ± 1.4 *	< 0.001	75.8 ± 2.5 *#	11.3 ± 0.4 *#	< 0.001

Data are represented as means ± SEM. AP, alkaline phosphate; BUN, blood urea nitrogen; Ca, calcium; Ca x P, calcium x phosphorus product; Gr., group; P, phosphorus. * Vs. Baseline; p < 0.05. ** Vs. Baseline; p < 0.001. # Vs. Week 9; p < 0.05. ## Vs. Week 9; p < 0.001.

## Discussion

In the present study, we have shown that echocardiographic cIB is a useful tool for the non-invasive, quantitative monitoring of progression and subsequent spontaneous regression of aortic valve calcifications in a rat model of diet-induced renal failure. We validated this technique by comparison with histology.

### CAVD and its relationship with renal failure

CAVD accounts for 70% of all valvular heart diseases and evolves to significant aortic valve stenosis in about 10% of patients. In this population, the outcome is poor as there is a significant 5-year increased risk of progression to heart failure or death [15]. Hence, there is a need for medical treatment of CAVD. However, reversal of end-stage macrocalcification is deemed difficult [3]. To date, medical therapies have failed to demonstrate a significant effect on advanced valvular calcifications [4]. In animal studies, only early preventive medical interventions have shown to be of interest [9].

Renal failure is a major risk factor for cardiovascular calcifications and CAVD due to secondary hyperparathyroidism and a disturbed mineral metabolism with increased Ca × P [2]. In chronic kidney disease, approximately 50% of patients will develop CAVD at an accelerated rate, leading to increased morbidity and mortality [16,17]. Small animal models such as rats have become increasingly important to study renal failure and CAVD [9]. Shuvy et al. have previously investigated a rat model of adenine and high-phosphate diet-induced renal failure and subsequent CAVD [10]. Interestingly, in this study discontinuation of the diet led to a progressive recovery of the renal function after 2 weeks and marked spontaneous improvement of the aortic valvular calcifications after 12 weeks [10]. The observed reversibility of CAVD might offer an opportunity for the development of new medical treatments.

### Imaging of aortic valve calcifications

Calcification is an independent predictor of morbidity and mortality in CAVD [2,3]. Quantification of calcifications in CAVD with in vivo imaging tools could be helpful for the monitoring of disease progression and potential regression through medical interventions. Although the reference approach, the use of cardiac CT is limited due to irradiation exposure of patients. Repetitive evaluation by cardiac CT is thus hampered. Conventional echocardiography primarily makes use of specular reflections and Doppler ultrasound to investigate valve morphology and function [18]. Aortic valve calcification is rather regarded as a categorical variable with the current qualitative methodology [19]. However, by analyzing backscatter reflections generated by the interaction of ultrasound with small tissue structures, it is possible to quantitatively extract information related to the valve’s composition [12,20,21]. IB has previously been extensively validated in the myocardium as a surrogate marker of fibrosis and for the differentiation of atherosclerotic lesions [22,23]. We have previously shown that with IB calcific deposits of the aortic valve can be quantitatively and reproducibly assessed in rats [7,8]. Others have used IB for the quantitative detection of aortic valve calcifications in vitamin D treated rabbits, which could be delayed with ramipril [24,25]. Preliminary clinical studies have demonstrated that higher backscatter scores are seen in sclerotic compared to non-sclerotic aortic valves, with a further increase in stenotic valves [26,27]. Backscatter scores of the aortic valve directly correlated with subjective scoring and with transvalvular pressure gradients in patients [13].

### Integrated backscatter analysis of aortic valve calcifications

In the present study, we have shown that cIB can detect a significant progression of CAVD after 9 weeks in rats with renal failure, compared to baseline and controls of the same age. Twelve weeks after discontinuation of the adenine diet, the cIB of the aortic valve returned to values similar to controls, suggesting a regression of the calcifications. This confirms the validity of cIB for the quantitative evaluation of aortic valve calcifications [7,8,24]. Moreover, it corroborates the findings of Shuvy et al. in a similar model. In that particular study, quantitative CT was used for assessing CAVD. However, the amount of calcifications on histology and echocardiography were not provided [10]. In our study, there was a significant correlation between cIB and the histological Ca Area for the aortic valve. Moreover, there was a significant inter- and intra-observer reproducibility of cIB values. Global cIB values (ROI_AVGLOB_) and mean cIB values (ROI_AVTOT_) also correlated significantly. This implies that the global cIB values can be used, potentially simplifying the technique for daily practice.

Blood results showed a significant decrease of the renal function during adenine feeding in group 1 compared to controls, associated with a significant increase of P, Ca × P, AP and osteocalcin. After diet cessation in group 1, P, Ca × P and AP normalized at 21 weeks. Interestingly, in our study there was only a minor improvement of the renal function after diet cessation in group 1, in contrast to the complete recovery seen in the study of Shuvy et al. [10]. This could be due to a difference in susceptibility of the rat strain (Wistar versus Sprague–Dawley). Our results confirm a previous study by Okada et al., suggesting irreversible renal failure after 4 weeks of treatment with adenine (0.75%) [28]. As a regression of CAVD was seen in spite of the remaining elevated creatinine values, it seems that the metabolic changes induced by renal failure and secondary hyperparathyroidism may be the major mediators of CAVD, rather than renal failure per se [10].

The elevation and subsequent regression of AP might confirm a role of increased bone turnover with mobilisation of Ca and P in secondary hyperparathyroidism-associated CAVD [29]. Osteocalcin levels remained high at the end of the study in group 1, even 12 weeks after diet cessation. This calcification inhibitor might be produced by differentiated osteoblast-like cells in the calcifying cardiovasculature in an attempt to further prevent and/or reduce calcifications [4]. In the adenine-fed group 1, there was a weight loss as previously described [30], with a reduction in EF, CO and transvalvular PG on echocardiography. However, LV performance returned to normal after diet cessation in group 1.

### Future perspectives

Backscatter software is now widely available on recent echocardiography packages, which makes this tool feasible for clinical application. In daily practice, cIB may be used for the detection of early aortic valve calcifications, for the assessment of disease severity and progression as well as for evaluating the effectiveness of potential medical interventions in prospective and longitudinal trials.

## Conclusions

Calibrated integrated backscatter is a promising, non-invasive echocardiographic tool to quantitatively monitor progression and regression of aortic valve calcifications in a rat model of diet-induced renal failure, as validated by comparison with histology. This technique might become useful for assessing CAVD during targeted therapy.

## Abbreviations

A: Peak diastolic late velocity; AP: Alkaline phosphate; AV: Aortic valve; BP: Blood pool; BUN: Blood urea nitrogen; Ca: Calcium; Ca x P: Calcium x phosphorus; Ca Area: Calcified area; CAVD: Calcific aortic valve disease; cIB: Calibrated integrated backscatter; CO: Cardiac output; CT: Computed tomography; dB: Decibel; Dec Time: Deceleration time; E: Peak diastolic early velocity; EF: Ejection fraction; Gr.: Group; IB: Integrated backscatter; LA: Long-axis; LV: Left ventricle; LVEDV: LV end-diastolic volume; LVESV: LV end-systolic volume; LVOT: LV outflow tract; P: Phosphorus; PG: Pressure gradient; ROI: Region of interest; ROIAVGLOB: Region of interest aortic valve, global; ROIAVLA region of interest: Aortic valve, long-axis; ROIAVSA region of interest: Aortic valve, short-axis; ROIAVTOT: Region of interest aortic valve, total; SA: Short-axis

## Competing interests

The authors declare that they have no competing interests.

## Authors’ contributions

BR, SD, GVC and BC conceived the study design and planned the coordination. BR, KG, and GB followed the animals’ condition daily, acquired echocardiographic measurements and analyzed the ultrasound data. JS, ED, PD and IR prepared and analyzed the histological data. JDN and JS carried out the biochemistry. BR, KG, SD and BC interpreted the data and wrote the manuscript. PD, GVC, PL, SD and BC revised the manuscript critically for important intellectual content. All authors have given approval of the final version of the manuscript to be published.
